# Substrate oxidation enhances the electrochemical production of hydrogen peroxide

**DOI:** 10.1016/j.cej.2019.05.165

**Published:** 2019-10-15

**Authors:** Jonghun Lim, Michael R. Hoffmann

**Affiliations:** Linde + Robinson Laboratories, California Institute of Technology, Pasadena, CA 91125, United States

**Keywords:** Electrocatalysis, Hydrogen peroxide, Anodic decomposition of H_2_O_2_, Organic electron donors

## Abstract

Hydrogen peroxide (H_2_O_2_) is electrochemically produced via oxygen (O_2_) reduction on a carbon cathode surface. In order to enhance the production of H_2_O_2_, anodic loss pathways, which significantly reduce the overall H_2_O_2_ production rate, should be inhibited. In this study, we investigate the effects of organic electron donors (*i.e.*, typical chemical contaminants) on the anodic loss pathways of H_2_O_2_ in a single-cell electrochemical reactor that employs an anode composed of TiO_2_ over-coated on a mixed-metal oxide ohmic contact catalyst, Ir_0.7_Ta_0.3_O_2_, deposited on a Ti-metal that is coupled with a graphite rod cathode in a sodium sulfate (Na_2_SO_4_) electrolyte that is saturated with oxygen (O_2_). Organic electron donors are shown to enhance the electrochemical production of H_2_O_2_, while simultaneously undergoing oxidative degradation. The observed positive effect of organic electron donors on the electrochemical production of H_2_O_2_ is due in part to a preferential adsorption of organic substrates on the TiO_2_ outer layer of the anode. The sorption of the organic electron donors inhibits the formation of surficial titanium hydroperoxo species (≡Ti-OOH) on the anode surface. The organic sorbates also act as scavengers of surface-bound hydroxyl radical ≡Ti-OH. As a result, the decomposition of H_2_O_2_ on the anode surface is significantly reduced. The cathodic production rate of H_2_O_2_ at low pH is enhanced due to proton coupled electron transfer (PCET) to O_2_, while the anodic decomposition of H_2_O_2_ is inhibited due to electrostatic interactions between negatively-charged organic substrates and a positively-charged outer surface of the anode (TiO_2_ pH_zpc_ = 5.8) at low pH.

## Highlights

• The electrochemical production of H_2_O_2_ is enhanced in the presence of organic electron donors (*i.e.*, pollutants).
• The oxidation of organic substrates prevents the anodic decomposition of H_2_O_2_.
• The production of H_2_O_2_ with simultaneous organic pollutants degradation is more efficient under acidic conditions.

## Graphical abstract

**Figure:**
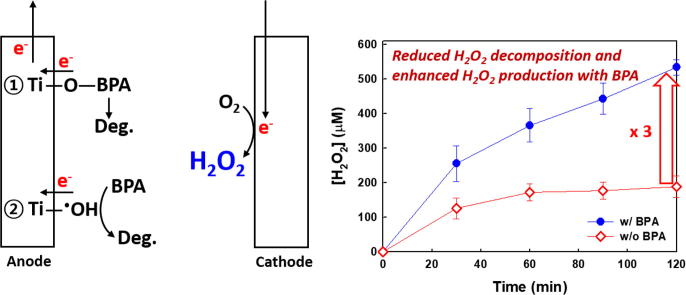

## 1 Introduction

Hydrogen peroxide (H_2_O_2_) is often used as an eco-friendly oxidant because it is reduced to water as an electron acceptor and readily decomposes to water and oxygen (O_2_) [1]. The high oxidation potential (*E*_0_ = +1.76 V_NHE_) of H_2_O_2_ allows for the direct oxidation of certain organic and inorganic electron donors and for the indirect oxidation by hydroxyl radical produced via the UV photolysis of H_2_O_2_ or by Fenton-reagent activation [2]. Hydrogen peroxide is also used in organic syntheses, liquid fuel rocket propulsion, disinfection, and environmental remediation [1]. Moreover, H_2_O_2_ is frequently used in advanced oxidation processes for water or air [3], [4], [5].

Hydrogen peroxide is normally produced by the anthraquinone method involving a multistep oxidation of 2-ethyl-9,10-dihydroxyanthracene and the subsequent hydrogenation of 2-ethylanthraquinone [6]. However, this method is not environmentally benign because hydrogen (H_2_) gas, organic solvents, and high energy inputs are required. The direct reaction between the H_2_ and O_2_ gas using metal-based catalysts (*e.g.*, Au or Pd/Au alloys) in acid or methanolic solutions has been investigated as an alternative method for H_2_O_2_ production but this method is also not environmentally and economically viable [7], [8] due to the explosion potential of the H_2_ and O_2_ gaseous mixture [9], [10]. In contrast, the electrochemical production of H_2_O_2_ via a two-electron transfer to O_2_ is relatively benign synthetic method since it takes place at low temperatures and pressures [11].

Two pathways are available for the electrochemical generation of H_2_O_2_: (1) reduction of O_2_ on an appropriate cathode (Eq. (1)) [11] and (2) oxidation of water on a suitable anode material (*e.g.*, an anode with a high overpotential for the OER) (Eq. (2)) [12].

(1) O_2_ + 2H^+^ + 2e^−^→H_2_O_2_

(2) 2H_2_O → H_2_O_2_ + 2H^+^ + 2e^−^

Anodes and cathodes optimized for H_2_O_2_ production have been developed [13], [14], [15], [16]. However, the electrochemical synthesis of H_2_O_2_ is still limited by the decomposition of H_2_O_2_ on the surfaces of both the cathode (Eqs. (3), (4)) and anode (Eqs. (5), (6), (7)) [17].

(3) H_2_O_2_ + e^−^+H^+^→H_2_O+^•^OH

(4) H_2_O_2_ + 2H^+^+2e^−^→2H_2_O

(5) H_2_O_2_ → HO_2_^•^+H^+^+e^−^

(6) H_2_O_2_ → O_2_ + 2H^+^+2e^−^

(7) H_2_O_2_ + HO^•^→HOO^•^+H_2_O

In order to improve the overall yield of H_2_O_2_ during the electrochemical production, the decomposition of H_2_O_2_ should be substantially reduced. For example, carbon-based cathodes have been used since they inhibit the cathodic decomposition of H_2_O_2_ [18]. However, the strategy for preventing the anodic decomposition of H_2_O_2_ has received little attention.

In this study, we investigate the effect of organic electron donors on the electrochemical production of H_2_O_2_ as a method to inhibit the anodic decomposition of H_2_O_2_. Our hypothesis is that the production of H_2_O_2_ should be enhanced in the presence of organic electron donors at a constant cathodic potential. Organic substrates should prevent the anodic decomposition of H_2_O_2_ with respect to further anodic oxidation to superoxide and oxygen. The impact of experimental variables including applied voltage, pH, and probe reagents on the production of H_2_O_2_ in the presence of specific organic electron donors is explored.

## 2 Materials and methods

### 2.1 Materials and chemicals

A dimensionally-stable anode consisting of Ir_0.7_Ta_0.3_O_2_ formed during *in situ* spray pyrolysis of precursor reagents on a heated titanium metal substrate and over-coated with TiO_2_ was used (TiO_2_/Ir_0.7_Ta_0.3_O_2_/Ti). This composite anode formulation has been shown to be active with respect to both the chlorine and oxygen evolution reactions [19]. The aqueous-phase precursor solutions were composed of 3.5 mM IrCl_3_ and 1.5 mM TaCl_5_ in isopropanol for formation of the Ir_0.7_Ta_0.3_O_2_ layer, while the TiO_2_ overcoating layer was formed using a 25 mM titanium-glycolate solution for deposition of the overcoating TiO_2_ layer was deposited by spray coating of the solution directly on to Ti foil heated to 300 °C. The resulting film was annealed at 500 °C for 10 min. These procedures were repeated to reach a targeted mass loading. Upon achieving the desired mass loading, the final composite was annealed at 500 °C for 1 h. Chemical reagents used in this study were as follows: sodium sulfate (Na_2_SO_4_, Sigma-Aldrich), bisphenol A (BPA, Aldrich), phenol (J. T. Baker), 4-chlorophenol (4-CP, Sigma-Aldrich), coumarin (Sigma), potassium bis(oxalato)-oxotitanate (IV) dihydrate (K_2_[TiO(C_2_O_4_)_2_]∙2H_2_O, Alfa Aesar), sulfuric acid (H_2_SO_4_, J. T. Baker), hydrogen peroxide (H_2_O_2_ (30 wt%), Sigma-Aldrich). All chemical reagents were used as received without any purification. Deionized water was used as solution and prepared by a Millipore system (≥18 MΩ^•^cm, Milli-Q).

## 3 Electrochemical experiments

A three-electrode configuration including a working electrode (graphite rod, diameter 6 mm), a counter electrode (TiO_2_/Ir_0.7_Ta_0.3_O_2_/Ti), and a reference electrode (Ag/AgCl) was employed in a single compartment cell with a working volume of 25 mL. The background electrolyte was a 60 mM aqueous solution of Na_2_SO_4_. The optimum concentration of Na_2_SO_4_ was found to be 60 mM in terms of electrochemical efficiency (Fig. S1). As a consequence, the electrochemical reactions were primarily run in the 60 mM Na_2_SO_4_ electrolyte solution. The distance between the anode and cathode was 13 mm. During testing for the simultaneous electrochemical production of H_2_O_2_ and the concomitant degradation of organic substrates, a constant cathodic potential was applied to the electrodes using a computer-controlled potentiostat (SP-50, BioLogic). An aliquot of a substrate stock solutions (BPA, phenol, and 4-CP) was added to the electrolyte to give an establish a pre-set concentration of the target substrate. The initial pH was adjusted to a set value using either 1.0 M HClO_4_ or 1.0 NaOH solutions. Oxygen was purged in to the reactor for 30 min before application of a constant potential and then continuously purged during the course of electrolysis. Nitrogen (N_2_) gas purging of the aqueous solutions was carried out when low concentrations of dissolved oxygen were required. Aliquots of 1 mL were intermittently withdrawn from the reactor using a 1-mL pipet and were transferred into a glass vial without filtration for the analysis of the concentration of H_2_O_2_ and organic pollutants. Cyclic voltammetry (CV) data were collected in the Na_2_SO_4_ solution in the potential range of −0.8 to 0.0 V at a scan rate of 50 mV s^−1^.

## 4 Analysis

The concentrations of BPA, phenol, and 4-CP were quantitatively analyzed using a high performance liquid chromatograph (HPLC, Agilent 1100 series) equipped with a Zorbax XDB column. The HPLC measurement was carried out using a binary mobile phase of acetonitrile and phosphoric acid (30%:70% for BPA and 10%:90% for phenol and 4-CP). Chloride produced by 4-CP degradation was monitored using an ion chromatograph (IC, Dionex, USA) with an anion-exchange column (Ionpac AS 19). The total organic carbon (TOC) was analyzed using a TOC analyzer (Aurora TOC). The production of ^•^OH was monitored using coumarin as a chemical trap of ^•^OH. Coumarin is oxidized by hydroxyl radical to form 7-hydroxycoumarin [20]. The hydroxylated product, 7-hydroxycoumarin, was quantified by measuring the fluorescence emission intensity at λ_em_ = 456 nm after excitation at λ_ex_ = 332 nm·H_2_O_2_ was determined spectrophotometrically using potassium titanium (IV) oxalate [21]. The absorbance at 400 nm (ε = 9351 mol^−1^ cm^−1^) was measured using a UV/Visible spectrophotometer (Nanodrop 2000c).

## 5 Results and discussion

### 5.1 Simultaneous H_2_O_2_ production and BPA degradation

**Fig. 1:**
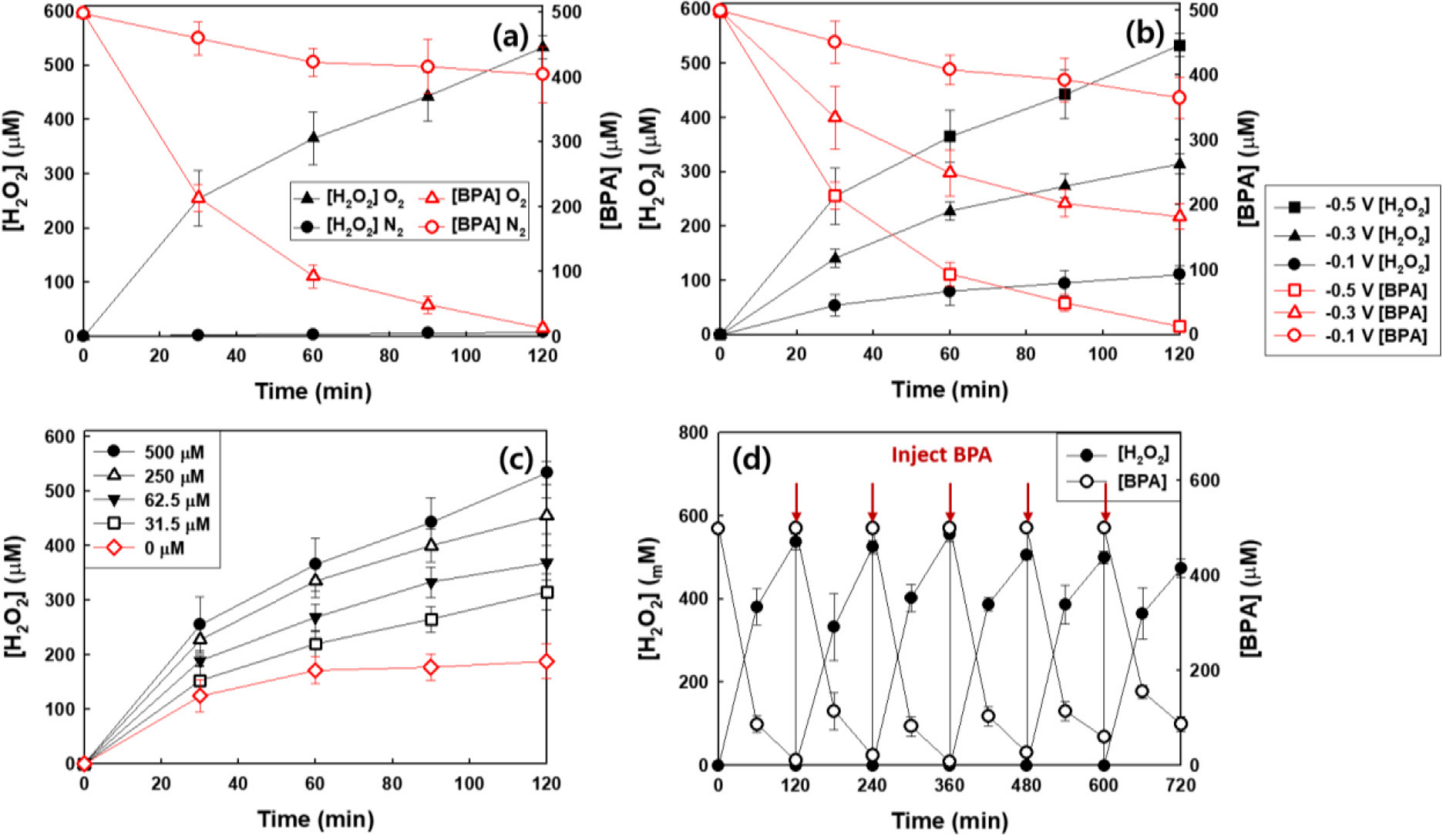
Time profiles of simultaneous H_2_O_2_ production and BPA degradation (a) under O_2_-or N_2_-purged condition and (b) under different applied cathodic voltage. (c) Effect of BPA concentration on the production of H_2_O_2_. (d) Repeated runs for H_2_O_2_ production and the concurrent BPA degradation. ([Na_2_SO_4_]_0_ = 60 mM; [BPA]_0_ = 500 µM (for a, b, and d); *E*_cell_ = −0.5 V (for a, c, and d); pH_i_ = 3.0; continuously O_2_^−^ or N_2_-purged (for a)).

Fig. 1a demonstrates that the production of H_2_O_2_ proceeds simultaneously with BPA degradation in the Na_2_SO_4_ electrolyte at a constant potential under O_2_-purging. Under N_2_ purging the production of H_2_O_2_ was negligibly low as shown in Fig. 1a. Given this result it is clear that H_2_O_2_ is primarily produced via O_2_ reduction at the cathode (Eq. (1)) [11]. This result was confirmed using cyclic voltammetry (Fig. S2). A reduction peak appeared near −0.4 V vs. Ag/AgCl in the presence of O_2_ is attributed to the reduction of O_2_ leading to the formation of H_2_O_2_ [22], [23]. The reduction peak at −0.4 V disappeared in the N_2_-purged solution as observed previously [11], [22]. The degradation of BPA was also reduced in the absence of O_2_ (Fig. 1a). Fig. 1b shows the production of H_2_O_2_ coupled with BPA degradation as a function of the applied potential. The efficiencies for the production of H_2_O_2_ and degradation of BPA were increased with increasing the applied potential. H_2_O_2_ was not produced in the absence of an external potential bias, whereas the [BPA] was slightly reduced (Fig. S3). This result is most likely due to the adsorption of BPA on to the surface of anode at pH 3. Fig. 1c compares the production of H_2_O_2_ as a function of the BPA concentration. The electrochemical generation of H_2_O_2_ was increased with an increasing concentration of BPA. In particular, H_2_O_2_ was continuously produced in the presence of BPA, whereas its generation reached an apparent steady-state level in the absence of BPA after 1 h of electrolysis. This steady-state is achieved due to the *in situ* decomposition of the H_2_O_2_ [23]. During repeated electrolytic cycles, a loss of the activity for BPA degradation and H_2_O_2_ production was not observed four catalytic cycles. However, cycling for more than four cycles resulted in a small loss of activity (Fig. 1d). This result can be ascribed to active site blocking on the electrode surface due to the adsorption of BPA and its reaction product intermediates generated during BPA degradation.

### 5.2 Influence of BPA on H_2_O_2_ decomposition

**Fig. 2:**
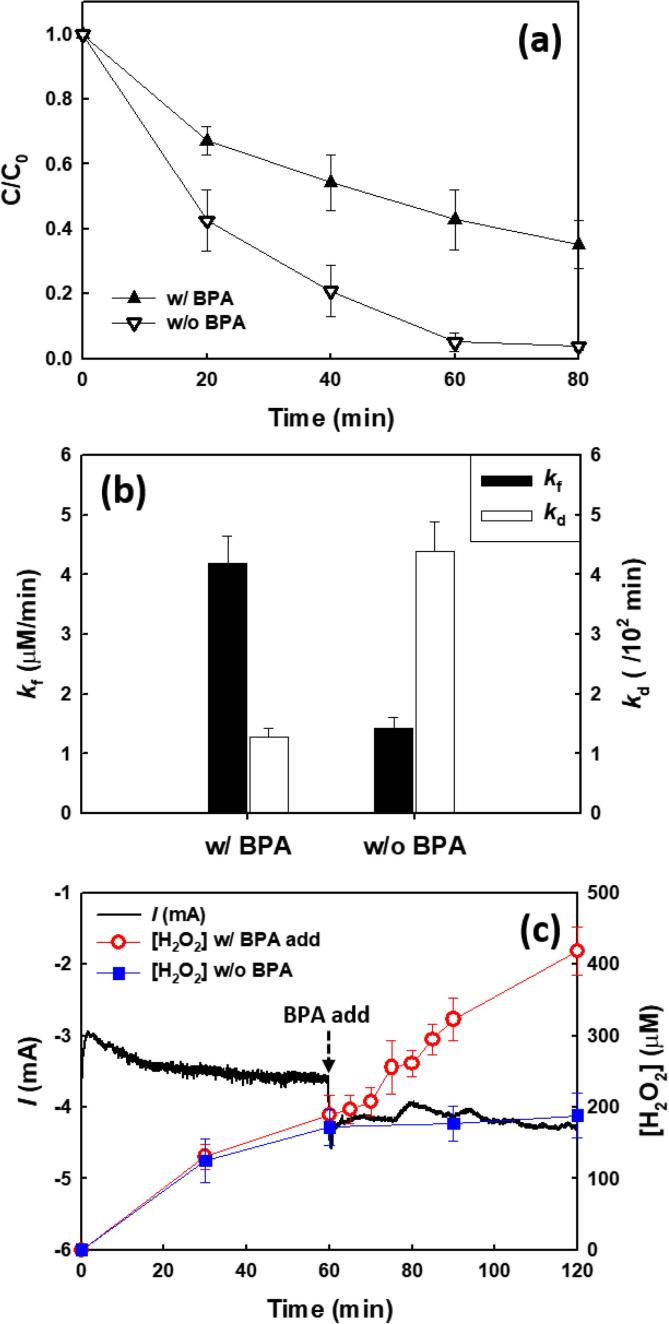
(a) Decomposition of H_2_O_2_ and (b) formation rate constant (*k*_f_) and decomposition rate constant (*k*_d_) for H_2_O_2_ in the presence and absence of BPA. (c) Effect of BPA on H_2_O_2_ production and cell current. BPA (1 mM) was added to the electrolyte (as indicated by arrows) in the course of electrolysis. ([Na_2_SO_4_]_0_ = 60 mM; [BPA]_0_ = 500 µM (for a and b); [H_2_O_2_]_0_ = 5 mM (for a and b); *E*_cell_ = −0.5 V; pH_i_ = 3.0; continuously O_2_-purged).

The effects of BPA on the kinetics of decomposition of H_2_O_2_ were determined by following the change in concentration of 5 mM of hydrogen peroxide in the electrolyte solution in the presence and absence of BPA (Fig. 2a). The decomposition of H_2_O_2_ in the absence of BPA was faster than that observed in the presence of BPA. Although the decomposition of H_2_O_2_ was significantly reduced at E_app_ = 0.0 V compared to E_app_ = −0.5 V, it was also found to be faster in the absence of BPA as shown in Fig. S4. The rate constant for H_2_O_2_ formation and decomposition were treated in terms of zero-order kinetics for production and first-order kinetics for decay, respectively [17]. The formation rate was increased and the decomposition rate was reduced in the presence of BPA compared to the absence of BPA (Fig. 2b). To further clarify the effect of BPA on the production of H_2_O_2_, excess BPA (1 mM) was added into the electrolyte during the course of electrolysis (after 1 h). The electrochemical production of H_2_O_2_ was enhanced by 55% (188 → 418 µM at 2 h) and the cathodic current was slightly increased (Fig. 2c) after adding BPA. These results clearly show that the presence of BPA as an anodic electrode donor offsets the *in situ* decomposition of H_2_O_2_ due to a net higher electrochemical rate of H_2_O_2_ formation via O_2_ reduction in the presence of BPA.

**Scheme 1:**
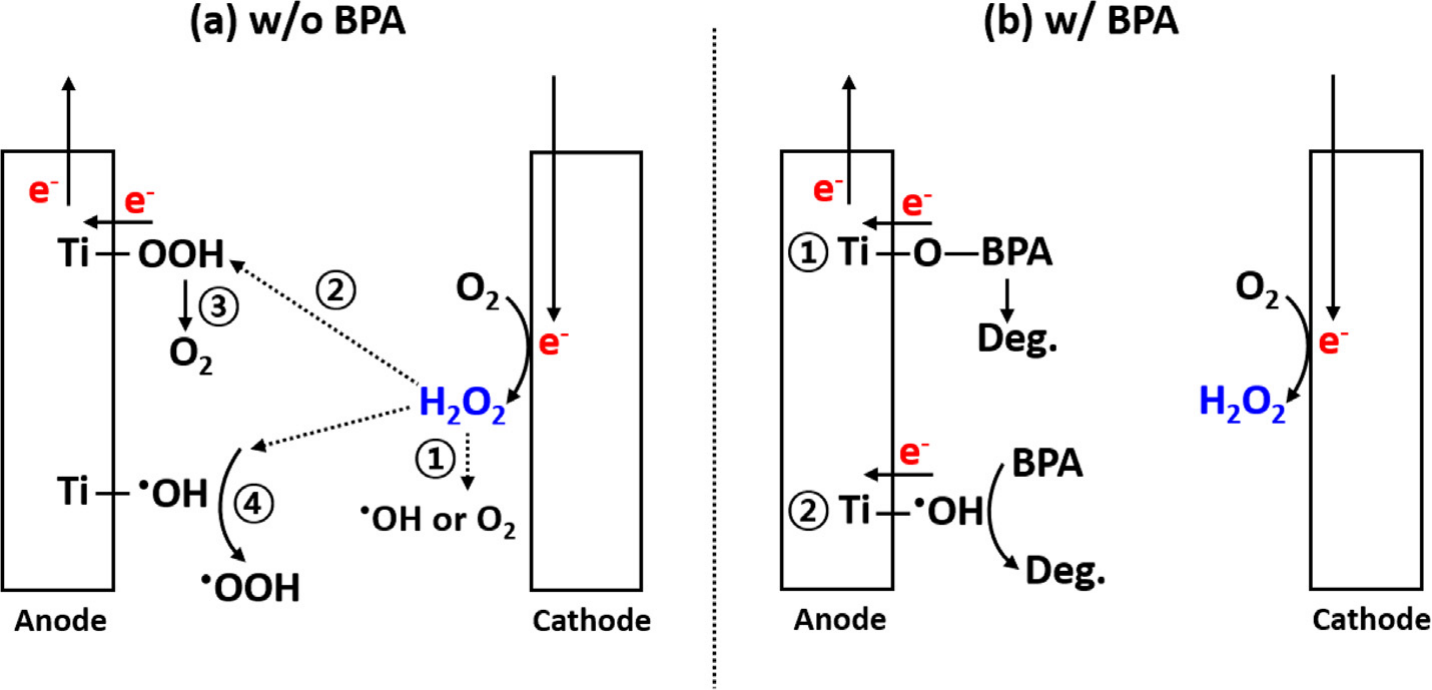
Schematic illustration of (a) anodic decomposition of *in situ* formed H_2_O_2_ in the absence of BPA and (b) production of H_2_O_2_ in the presence of BPA.

The electrochemically generated H_2_O_2_ could be decomposed on the surface of either the cathode or the anode (Scheme 1a). However, the cathodic decomposition of H_2_O_2_ can be excluded since most carbon-based cathodes including the graphite rod used in this study have been found to have low activities for H_2_O_2_ decomposition [18]. In the case of H_2_O_2_ decomposition on the anode, we expect to see the formation of hydroperoxo species (≡Ti-OOH) through the adsorption on the protective overlayer of TiO_2_ of the anode (reaction (2) in Scheme 1a) [17], [25]. We were able to confirm this possibility as shown in Fig. S4. However, we did not observe any noticeable difference and characteristic peaks related with hydroperoxo species in either the Raman or the XPS spectra of the anode before and after electrochemical reactions (*data not shown*). This may be due to the fact that surface-bound hydroperoxyl species are rapidly converted into HO_2_^•^ and O_2_ (Eqs. (5), (6) and reaction (3) in Scheme 1a) [24], [26]. Furthermore, H_2_O_2_ is decomposed by ^•^OH generated on the surface of the anode (Eq. (7) and reaction (4) in Scheme 1a) [27]. On the other hand, the anodic decomposition of H_2_O_2_ appears to be significantly inhibited in the presence of BPA (Scheme 1b). The formation of hydroperoxo species is most likely prevented due to the sorption of BPA is instead of H_2_O_2_ on the outer surface of the anode (reaction (1) in Scheme 1b and Fig. S3). In addition, BPA acts as a scavenger of ^•^OH (reaction (2) in Scheme 1b) given the second-order rate constant for ^•^OH + BPA ⟶ *k* = 6.9 × 10^9^ M^−1^ s^−1^. In comparison, the corresponding rate constant for ^•^OH + H_2_O_2_ ⟶ *k* = 3.2 × 10^7^ M^−1^ s^−1^ [28]. The competition between the two decomposition pathways in the presence of BPA at the anode surface has net effect of allowing for the solution phase concentration of H_2_O_2_ to increase with time until reaching a steady-state condition.

### 5.3 Effect of pH and electrolytes

**Fig. 3:**
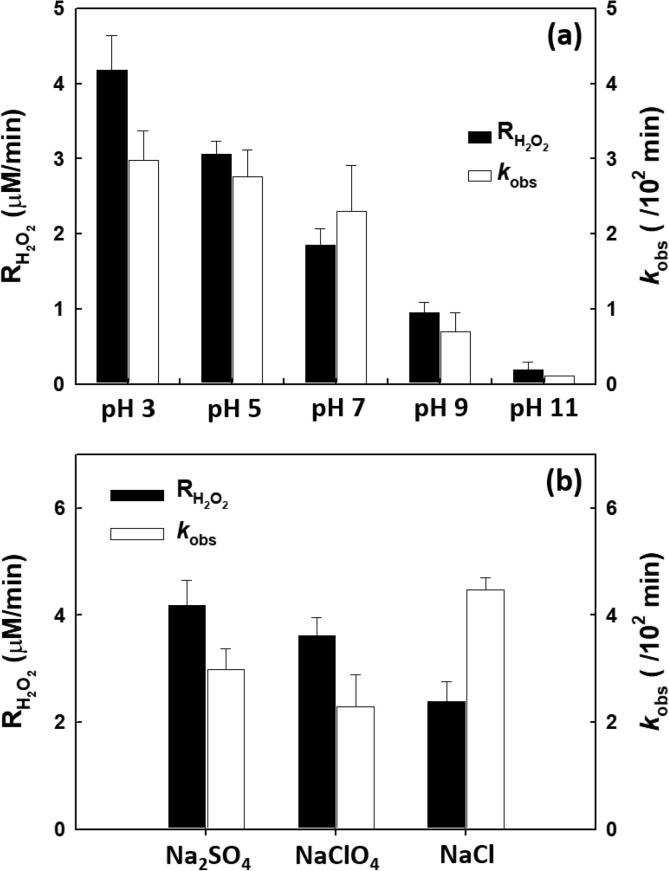
Effect of (a) initial pH and (b) electrolytes on the kinetic rate of simultaneous H_2_O_2_ production and BPA degradation. ([electrolyte]_0_ = 60 mM; [BPA]_0_ = 500 µM; *E*_cell_ = −0.5 V; continuously O_2_-purged).

The rates of H_2_O_2_ production and BPA degradation as a function of the initial pH of the electrolyte solution are shown in Fig. 3a. From this data, it is clear that the rate of H_2_O_2_ production decreased with increasing pH. These results may be due to the role of proton coupled electron transfer (PCET) to O_2_ (Eq. (1)) [29] leading on the electrochemical formation of H_2_O_2_ via O_2_ reduction at the cathode. The decrease in the rate of BPA degradation was minimal over the pH range of 3–7. However, the degradation rate decreased between pH 9 and 11. The increased in reaction rate with pH can be directly correlated with p*K*_a_ of BPA and the corresponding surface charge distribution of the TiO_2_ layer of the anode. The deprotonation of BPA (p*K*_a1_ = 9.6 and p*K*_a2_ = 10.2 yields the conjugate bases (HBPA^−^) and (BPA^2−^)) [30] results in an electrostatic repulsion of the anionic BPA species from the negatively charged TiO_2_ surface (pH_pzc_ (point of zero charge) ≈ 6.0 [31]) at higher pH. The electrostatic repulsion thus inhibits the adsorption of BPA on the anode surface at higher pH and thus the increased decomposition of H_2_O_2_. On the other hand, the adsorption of BPA on the anode surface facilitates the degradation of BPA under acidic and circum-neutral pH compared to alkaline pH. Furthermore, competitive adsorption of BPA on the anode surface inhibits the adsorption of H_2_O_2_ on the anode at lower pH resulting in the reduced anodic decomposition of H_2_O_2_ (*see* Fig. 2b). The pH-dependent results indicate that low pH conditions are more favorable for the proton-assisted electrochemical production of H_2_O_2_ coupled with the degradation of BPA.

The impact of the background electrolyte on the rates of H_2_O_2_ production and BPA degradation was examined as shown in Fig. 3b. The rates of H_2_O_2_ production and BPA degradation were slightly decreased in the NaClO_4_ electrolyte solution compared to our reference electrolyte Na_2_SO_4_. In contrast, the degradation of BPA was enhanced in the NaCl electrolyte solution compared to Na_2_SO_4_ [32]. The enhanced BPA degradation in a NaCl electrolyte is due to the anodic production of reactive chlorine species (RCS) (*e.g.*, chlorine radical (Cl^•^), dichloride radical anion (Cl_2_^•−^), hypochlorous acid (HOCl), and hypochlorite (OCl^−^)) oxidatively generated on the surface of anode in the presence of NaCl (Eqs. (8), (9), (10), (11), (12)) [33], [34].

(8) >Ti-OH + h^+^ → >Ti-^•^OH + e^−^

(9) >Ti-^•^OH + Cl^−^ → >Ti-OH + Cl^•^

(10) Cl^•^ + Cl^−^ → Cl_2_^•−^

(11) >Ti-^•^OH + Cl^−^ + H_2_O → >Ti-OH + HOCl + H^+^ + e^−^

(12) HOCl ⇌ OCl^−^ + H^+^

Given a sufficient applied potential, electron-hole pairs are formed and migration of a hole (h^+^) to a surface titanol group (>TiOH) leads to the formation of surface bound hydroxyl radical. However, the production of RCS has a negative effect on the electrochemical production of H_2_O_2_. The reactive chlorine species contribute collectively to the decomposition of H_2_O_2_ (Eqs. (13), (14), (15), (16)) [27]. In experiments described herein, the decomposition of H_2_O_2_ was accelerated at a high pH compared to low pH (Fig. S5). It is clear that H_2_O_2_ reacts faster with RCS (*e.g.*, ^−^OCl) under alkaline conditions compared to those at lower pH. For example, the bimolecular rate constant for reaction of H_2_O_2_ with hypochlorite (HOCl; p*K*_a_ = 7.6) (Eq. (12)) is substantially higher at high pH (7.5 × 10^3^ M^−1^ s^−1^) compared to circum-neutral pH (196 M^−1^ s^−1^) [35]. Furthermore, the RCS generated on the anode surface are reduced back to chloride on the cathode surface (Eqs. (17), (18), (19), (20)) [33]. Cathodic chloride reduction is competitive with the reduction of O_2_ leading to H_2_O_2_ production. Therefore, the rate of H_2_O_2_ production is significantly reduced in the presence of NaCl compared to Na_2_SO_4_ (Fig. 3b) consistent with the following set of reactions.

(13) H_2_O_2_ + Cl^•^ → HOO^•^ + H^+^ + Cl^−^

(14) H_2_O_2_ + Cl_2_^•−^ → HOO^•^ + H^+^ + 2Cl^−^

(15) H_2_O_2_ + HClO  →  H_2_O + O_2_ + H^+^ + Cl^−^

(16) H_2_O_2_ + OCl^−^ → Cl^−^ + O_2_ + H_2_O

(17) Cl^•^ + e^−^ → Cl^−^

(18) Cl_2_^•−^ + e^−^ → 2Cl^−^

(19) HClO + H^+^ + 2e^−^ → Cl^−^ + H_2_O

(20) OCl^−^ + H_2_O + 2e^−^ → Cl^−^ + 2OH^−^

### 5.4 Mechanism of BPA degradation

**Fig. 4:**
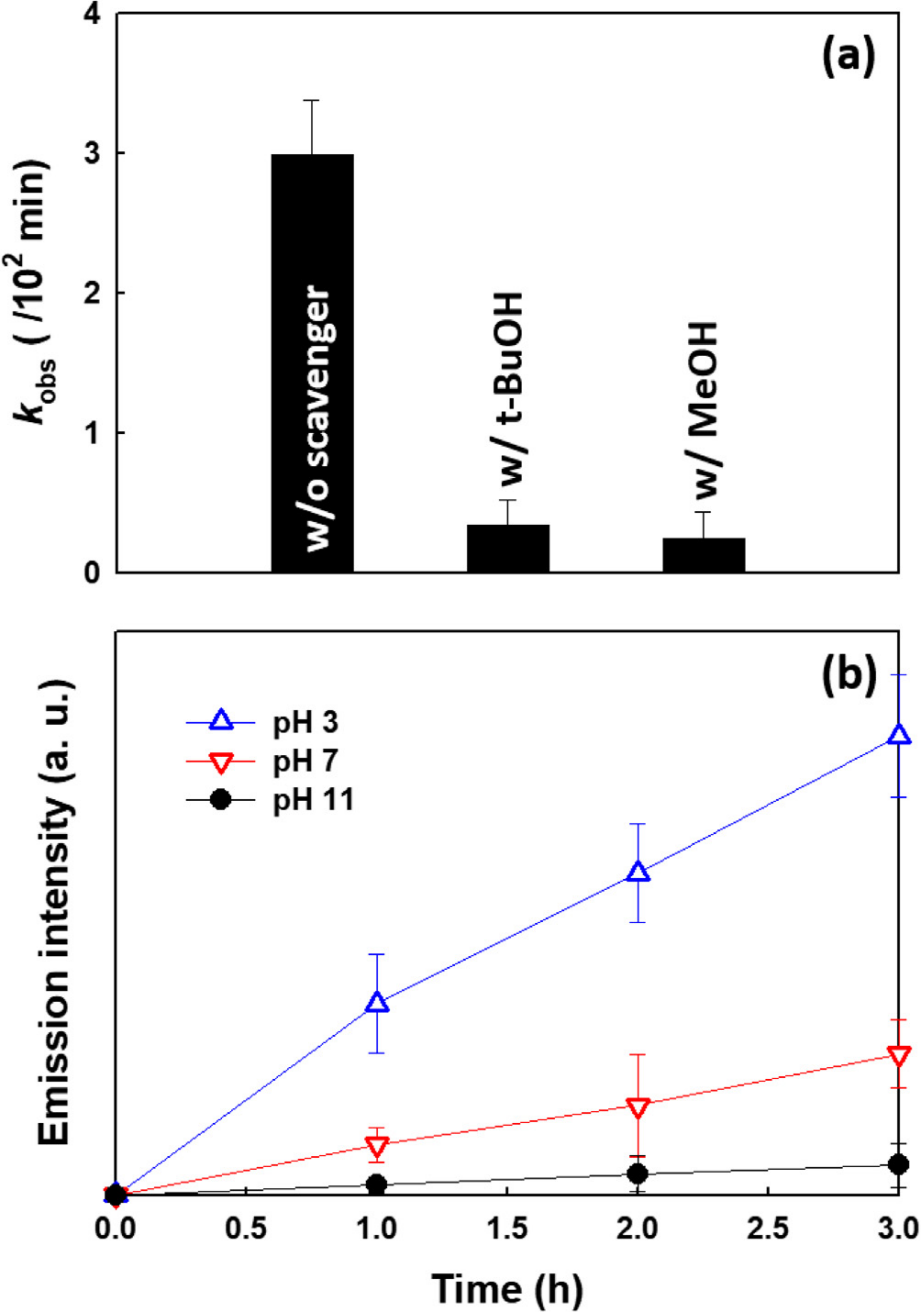
(a) Effect of t-BuOH and MeOH on BPA degradation. (b) Electrochemical production of coumarin-OH adduct (7-hydroxycoumarin). ([Na_2_SO_4_]_0_ = 60 mM; [BPA]_0_ = 500 µM (for a); [MeOH]_0_ = [t-BuOH]_0_ = 100 mM (for a); [coumarin]_0_ = 1 mM (for b); *E*_cell_ = −0.5 V; pH_i_ = 3.0 (for a); continuously O_2_-purged).

Direct electron transfer to a surface-trapped hole may contribute to BPA degradation. In order to confirm this possibility, the cathodic current was measured in the presence and absence of BPA under oxic O_2_ and then under anoxic N_2_ conditions (Fig. S6). Under these conditions, the cathodic current was slightly increased in the presence of BPA compared to the absence of BPA under both O_2_ and N_2_. These results imply that direct electron transfer to a surface-trapped hole provides a minor pathway for BPA degradation. The rate of BPA degradation in an NaClO_4_ electrolyte solution was found to be slightly reduced compared to the same reaction conditions in the Na_2_SO_4_ electrolyte (*see* Fig. 3b). This result suggests that SO_4_^•−^ may have been produced via anodic sulfate oxidation. Another possible oxidant in the system is the surface-bound hydroxyl radical (^•^OH) that is generated via surface titanol group oxidation (*i.e.*, >Ti-OH + h^+^) on the hydrated TiO_2_ surfaces of anode (Eq. (8)) [27]. To confirm the role of ^•^OH, BPA degradation was tested in the presence of *tert*-butanol (t-BuOH) and methanol (MeOH) as preferential radical scavengers of ^•^OH and SO_4_^•−^, respectively (Fig. 4a). t-BuOH and MeOH have similar bimolecular rate constants for reactions with ^•^OH (*e.g.*, 6.0 × 10^8^ M^−1^ s^−1^ and 9.7 × 10^8^ M^−1^ s^−1^, respectively); however, the rate constant for MeOH + SO_4_^•−^ ⟶ 3.2 × 10^6^ M^−1^ s^−1^ is higher than that for t-BuOH + SO_4_^•−^ ⟶ 4.0 × 10^5^ M^−1^ s^−1^ for the reaction with SO_4_^•−^ [36], [37]. t-BuOH and MeOH react with ^•^OH at similar rates, whereas MeOH reacts almost an order of magnitude faster with SO_4_^•−^ compared to t-BuOH. The quenching effects of t-BuOH (0.34 × 10^−2^ min^−1^) and MeOH (0.25 × 10^−2^ min^−1^) for BPA degradation are similar (Fig. 4a). This result demonstrates that BPA is mainly degraded by ^•^OH or surface-bound hydroxy radical, >TiOH^•^, which is consistent with the finding that BPA degradation in a Na_2_SO_4_ solution was similar to that in a NaClO_4_ solution (*see* Fig. 3b). If BPA is degraded by SO_4_^•−^, the quenching effect with MeOH should be greater than that with t-BuOH. However, the BPA degradation kinetics were not completely quenched. This result can be attributed to direct electron transfer to a surface-trapped hole by BPA leading to its degradation. The hypothesis is consistent with results showing a slight increase in the cathodic current in the presence of BPA compared to the absence of BPA under both O_2_ and N_2_ (*see* Fig. S6).

The generation of ^•^OH was further confirmed by using coumarin as a selective probe reagent for ^•^OH trapping. The hydroxylated products (7-hydroxycoumarin) generated by the reaction of coumarin with ^•^OH (^•^OH + coumarin  →  7-hydroxycoumarin) was quantified by monitoring the fluorescence emission [38]. Fig. 4b shows the pH-dependent electrochemical production of 7-hydroxycoumarin. The electrochemical production of 7-hydroxycoumarin was increased with decreasing pH, which demonstrates that the electrolytic degradation of BPA can be mainly ascribed to the facile production of ^•^OH as a primary oxidant produced on the surface of the anode at lower pH. This observation agrees with the data presented in Fig. 3a, which shows higher electrochemical activities for H_2_O_2_ production and BPA degradation under acidic and neutral pH compared to alkaline pH conditions. Even though a graphite rod normally has a low activity for catalyzing the decomposition of H_2_O_2_, the decomposition of H_2_O_2_ via this pathway (*see* Eq. (3)) cannot be ruled out. To test this possibility, the carbon cathode was replaced by a stainless steel cathode that has a lower activity for the electrochemical production of H_2_O_2_ than a graphite rod [39]. However, in spite of a significant reduction in the rate of H_2_O_2_ formation on the stainless steel cathode, the concomitant degradation of BPA was only slightly reduced compared to case of graphite rod cathode (Fig. S7). This result suggests that BPA degradation is initiated by surface-bound >TiOH^•^ radicals produced at the anode surface not by free ^•^OH radicals produced via H_2_O_2_ reduction at the cathode.

The pH change observed during electrolysis in the presence of BPA was completely different from that observed in the absence of BPA (Fig. S8). After applying an external bias potential (−0.5 V), the pH immediately increased from 5.8 to 7.5 in the absence of BPA due to the consumption of protons required for H_2_O_2_ production and then very slightly decreased. On the other hand, the pH was continuously reduced during the oxidation of BPA. This result is due to the formation of organic acids such as lactic, oxalic, fumaric, and glutaric acid [40]. Quinones and catechol were formed as reaction intermediates (Fig. S9a), which were, in turn, oxidized into organic acids. Despite the almost complete removal of BPA in 2 h, the TOC removal was only 66% after 4 h, although complete mineralized was achieve in 6 h (Fig. S9b).

**Fig. 5:**
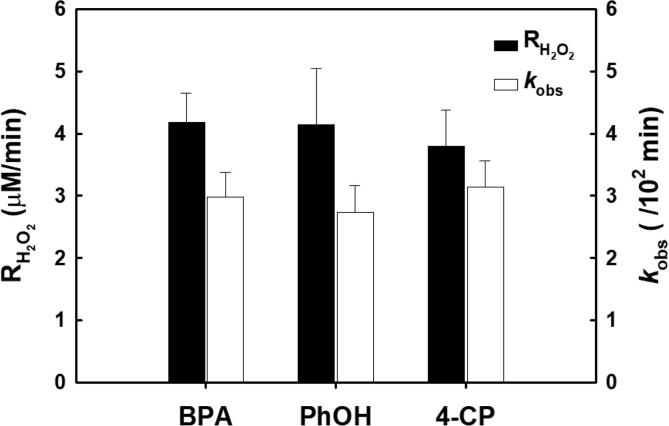
Simultaneous H_2_O_2_ production coupled with various phenolic organic pollutants degradation. ([Na_2_SO_4_]_0_ = 60 mM; [organic pollutants]_0_ = 500 µM; *E*_cell_ = −0.5 V; pH_i_ = 3.0; continuously O_2_-purged).

Phenol (PhOH) and 4-chlorophenol (4-CP) were electrolytically oxidized under identical conditions (Fig. 5) and were found to have similar rates degradation and H_2_O_2_ production compared to BPA. Even though reactive chlorine species were produced in the oxidation of 4-CP (Fig. S10), their effects on H_2_O_2_ production was minor compared to electrolysis in the NaCl electrolyte (*see* Fig. S5). Thus, we conclude that the RCS concentration produced during the electrolysis of 4-CP was low (<1 mM) [35].

## 6 Conclusions

Herein, we clearly demonstrate that positive effect of organic electron donors on the electrochemical production of H_2_O_2_. The organic substrates are preferentially adsorbed on the anode surface preventing the anodic oxidation of H_2_O_2_ formed on the cathode. Furthermore, the organic electron donors actively scavenge surface-bound hydroxyl radical (^•^OH), which also reacts competitively with H_2_O_2_. As a result, the oxidative decomposition of H_2_O_2_ is reduced resulting in the net accumulation of H_2_O_2_ during the electrolysis of organic pollutants.
