# Supplementary material for: Substrate oxidation enhances the electrochemical production of hydrogen peroxide
Source: Chem Eng J. 2019 Oct 15;374:958–64. doi: 10.1016/j.cej.2019.05.165 (PMC6686209; doi:10.1016/j.cej.2019.05.165)
Supplement: Supplementary data 1 [file mmc1.docx]

Appendix A. Supplementary Information

# **Substrate Oxidation Enhances the Electrochemical Production of Hydrogen Peroxide**

# Jonghun Lim^a^ and Michael R. Hoffmann^a^*

#

# ^a^ Linde + Robinson Laboratories, California Institute of Technology, Pasadena, California 91125, United States

# *Corresponding author.

E-mail: [mrh@caltech.edu](mailto:mrh@caltech.edu) (M. R. H.)


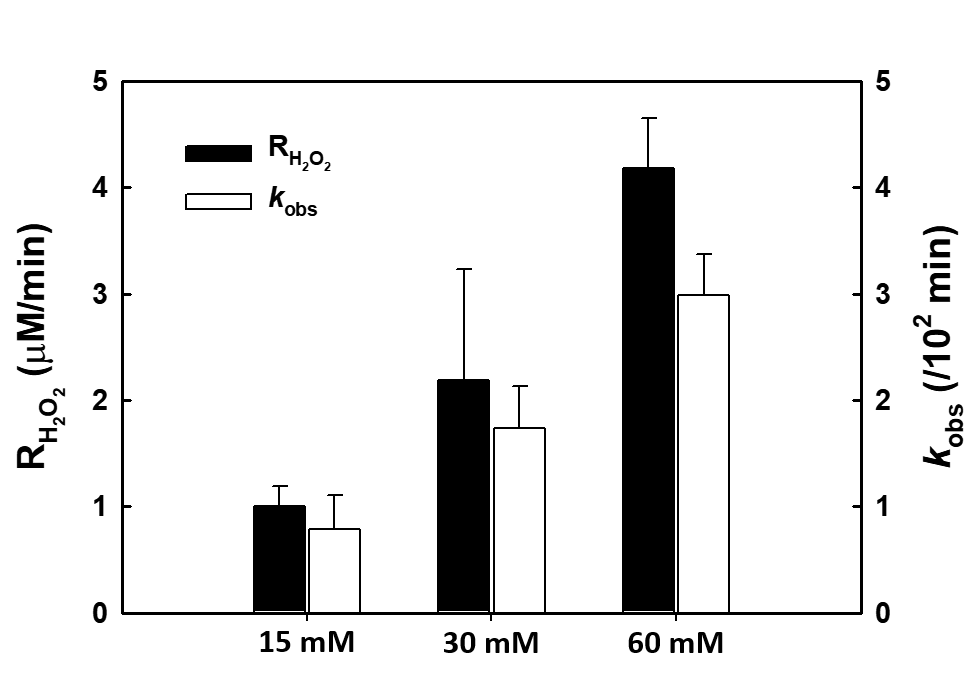


**Fig. S1.** Electrochemical BPA degradation and H_2_O_2_ production as a function of Na_2_SO_4_ concentration.


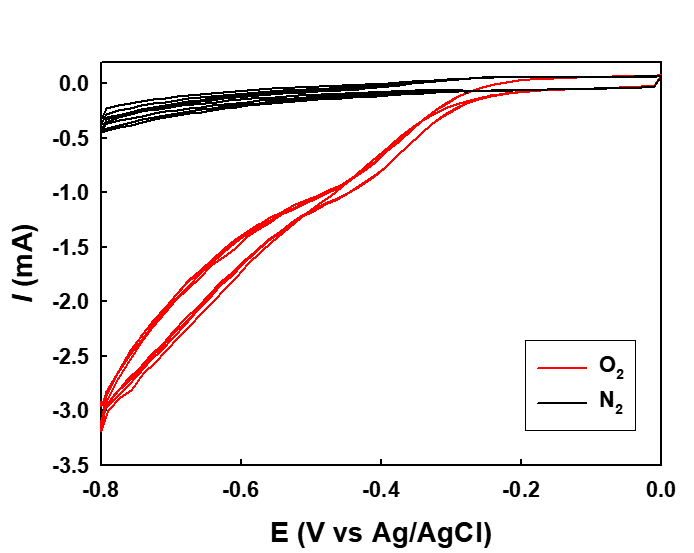


**Fig. S2.** Cyclic voltammograms under countinuously O_2_- or N_2_-purged condition. ([Na_2_SO_4_]_0_ = 60 mM; continuously O_2_- or N_2_-purged).


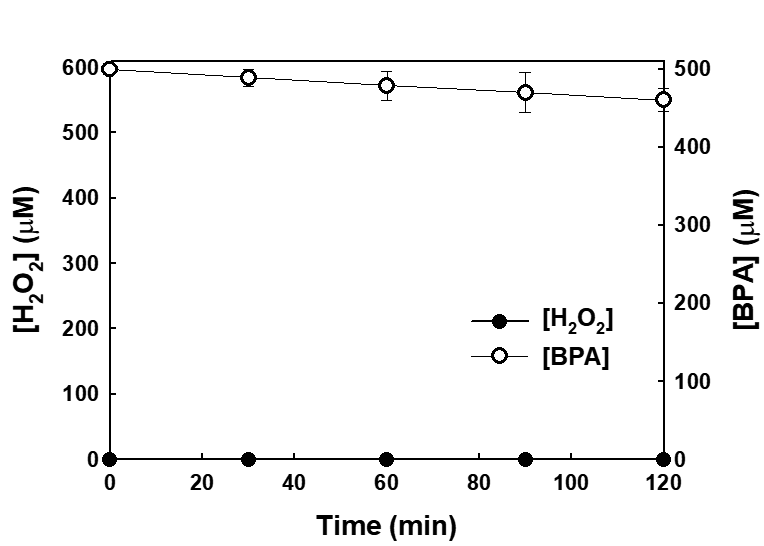


**Fig. S3.** Simultaneous H_2_O_2_ production and BPA degradation without an external bias potential (under 0 V). The experimental conditions were the same as those of Figure 1a except for applied cathodic voltage.


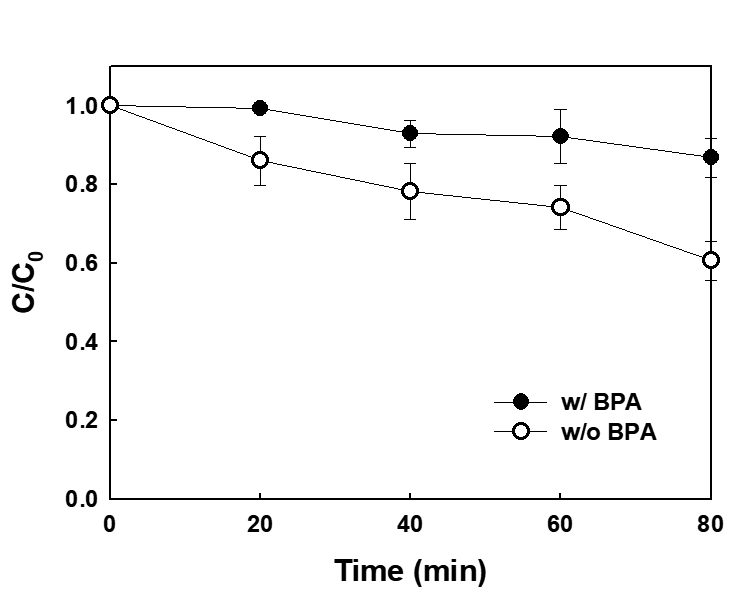


**Fig. S4.** H_2_O_2_ decomposition in the presence and absence of BPA under 0 V. The experimental conditions were the same as those of Figure 2a except for applied cathodic voltage.


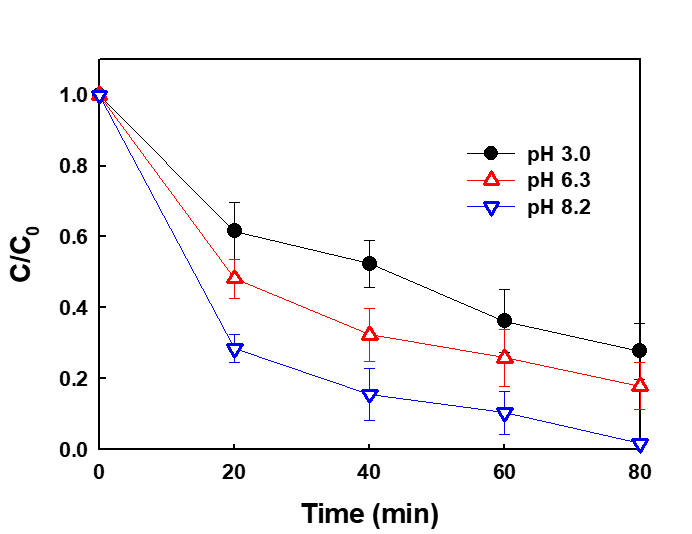


**Fig. S5.** Effect of initial pH on H_2_O_2_ decomposition in the presence of NaCl electrolyte. ([NaCl]_0_ = 60 mM; [H_2_O_2_]_0_ = 5 mM; *E*_cell_ = -0.5 V; continuously O_2_-purged).


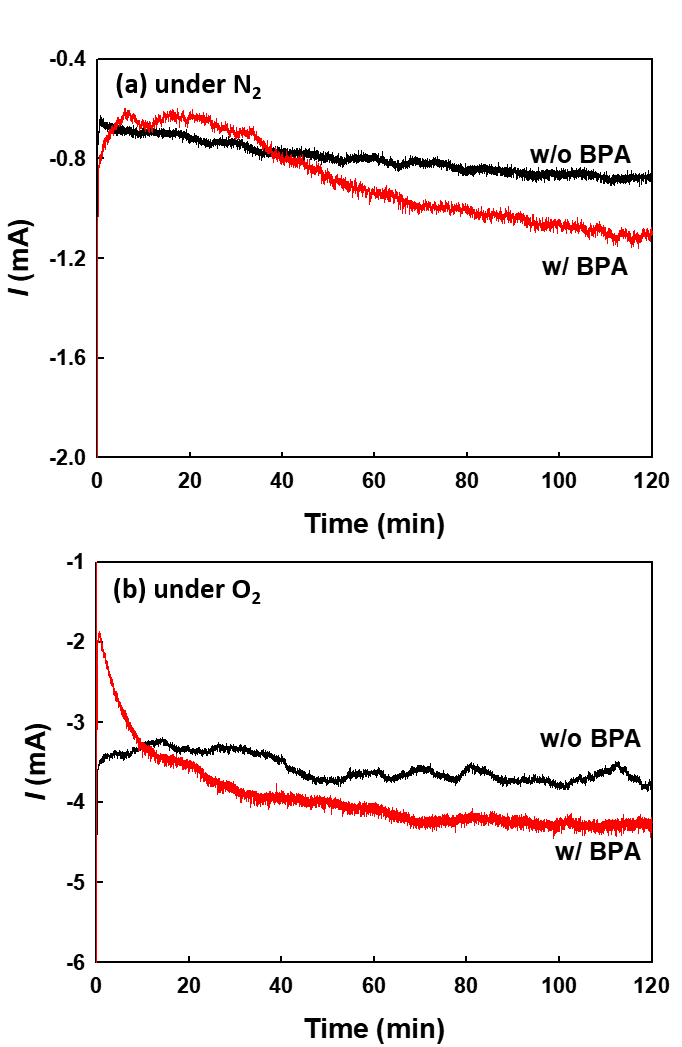


**Fig. S6.** Cathodic current generation in the presence and absence of BPA under continuously (a) N_2_- and (b) O_2_-purged condition. ([Na_2_SO_4_]_0_ = 60 mM; [BPA]_0_ = 500 µM; pH_i_ = 3.0).


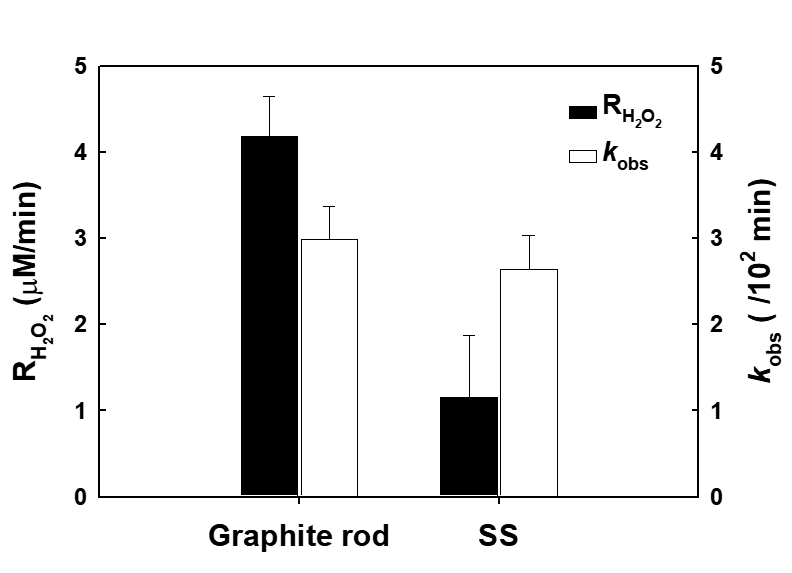


**Fig. S7.** Comparison of the electrochemical production of H_2_O_2_ and degradation BPA with graphite rod and stainless steel as a cathode. The experimental conditions were the same as those of Fig. 1a.


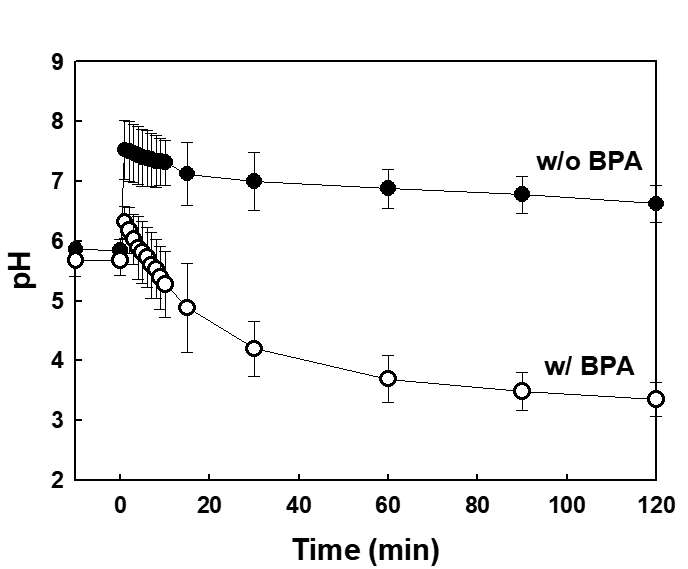


**Fig. S8.** Time profiles of pH change in the absence of presence of BPA. The experimental conditions were the same as those of Fig. 1a.


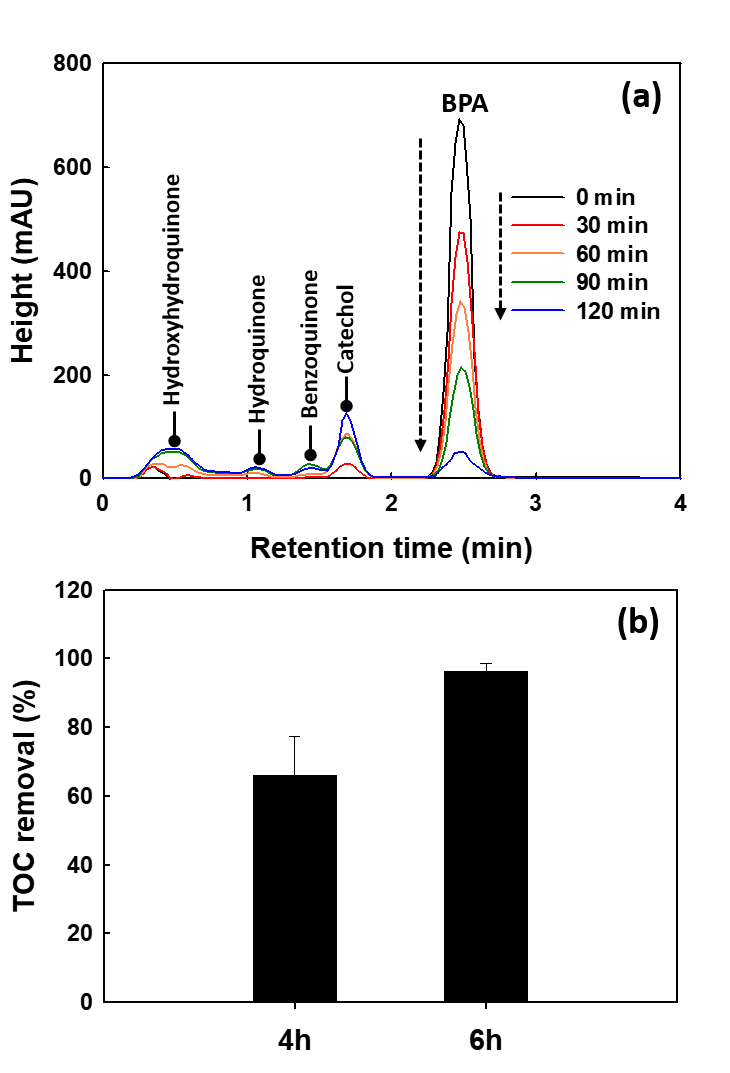


**Fig. S9.** (a) Intermediates produced from the BPA degradation. (b) TOC removal during 4 and 6 h electrolysis.


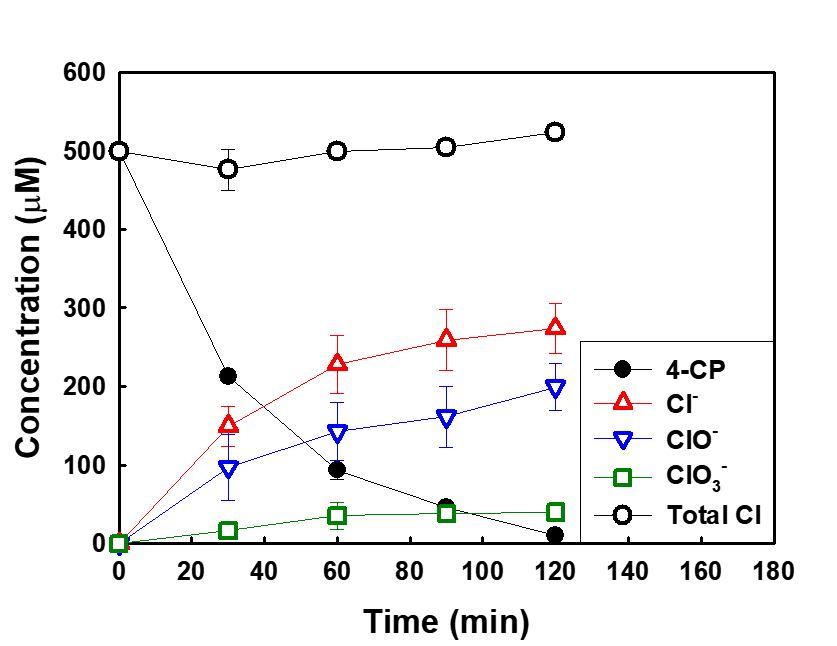


**Fig. S10.** Chlorine species generated from 4-CP degradation. The experimental conditions were the same as those of Fig. 5.
